# Editorial: Physical activity, motor competence and fitness: influences in the sociocultural context

**DOI:** 10.3389/fpsyg.2024.1397435

**Published:** 2024-05-01

**Authors:** Alessandro H. Nicolai Ré, Arto Laukkanen, Maria Teresa Cattuzzo

**Affiliations:** ^1^University of São Paulo, São Paulo, Brazil; ^2^Faculty of Sport and Health Sciences, University of Jyväskylä, Jyväskylä, Finland; ^3^Universidade de Pernambuco, Recife, Pernambuco, Brazil

**Keywords:** human development, environmental influences, public health, motor skills, sports

This Research Topic was conceived in the knowledge that the sociocultural context (SC) influences human development, requiring research into the specific reality of a given population. As such, there is a need to identify and report regularly contemporary correlates of physical activity (PA), motor competence (MC), and health-related fitness (HF) in different cultures/populations. With this aim in mind, FRONTIERS invited researchers to write articles identifying correlates and associations between PA, MC, and HF. This issue contains five articles to inform readers about (a) basic motor competencies in Slovak children, (b) weight status and socio-demographic disparities in children's motor and cognitive function, (c) gross motor skills of children through traditional games skills, (d) physical activity habits and their relationship with sociodemographic factors in Chilean adolescents, and (e) associations between socioeconomic status and physical activity of Chinese children and adolescents. Readers are strongly advised not to read only the paper most closely associated with their own specialist area, but to read all five papers in this Research Topic, as the methods and populations are not common.

The paper by Šiška et al. shows the level of motor competence of Slovak children and highlights gender and BMI as significant covariates for the results. Moss et al. found a significant association between healthy weight status and children's cognitive development, without an effect of gender. The research of Hussain and Cheong shows that playing traditional games favored the acquisition of fundamental motor skills, bringing an avenue for studying traditional game skills of/in different cultures. Fuentealba-Urra et al. show the level of physical activity in Chilean adolescents was below international recommendations and gender had the highest predictive effect. Results of the study by Ke et al. show that low-socioeconomic-status children were likely to practice lower levels of physical activity and different socioeconomic status indicators had different effects on students of different genders. In this Research Topic, research into the reality of different populations shows that gender may moderate the associations between SC and PA, MC, and HF. In addition, different SCs had different effects in different populations, justifying the need for public policies, interventions, and school physical education programs within the reality of a given population.

This Research Topic highlights the need of providing girls and boys with equal chances to engage in sports and other physical activities that are culturally significant, as this has a direct impact on active and healthy development. It is important to provide information for those who interact with children (teachers, parents, and other education and medical professionals), regarding physical development, physical activity, and health, as well as providing appropriate spaces and equipment for practicing a wide range of physical activities in schools and communities. In the research area, articles in this edition raise critical questions about how family and environmental circumstances impact children's active and healthy lives. Longitudinal studies, which have better potential to influence public health policies, should be used in future research to explore this topic, particularly in underprivileged populations.

## Author contributions

AR: Writing—original draft, Writing—review & editing. AL: Writing—original draft, Writing—review & editing. MC: Writing—original draft, Writing—review & editing.

